# Irradiation resistance mechanism of the CoCrFeMnNi equiatomic high-entropy alloy

**DOI:** 10.1038/s41598-020-79775-0

**Published:** 2021-01-12

**Authors:** Q. Xu, H. Q. Guan, Z. H. Zhong, S. S. Huang, J. J. Zhao

**Affiliations:** 1 Institute for Integrated Radiation and Nuclear Science, Kyoto University, Osaka, 590-0494 Japan; 2 Key Laboratory of Materials Modification by Laser, Ion and Electron Beams (Dalian University of Technology), Ministry of Education, Dalian, 116024 China; 3 School of Materials Science and Engineering, Hefei University of Technology, Hefei, 230031 China

**Keywords:** Materials science, Structural materials, Theory and computation

## Abstract

When face-centered cubic (FCC) metals and alloys with low stacking fault energy (SFE) are irradiated by high-energy particles or deformed at high speed, stacking fault tetrahedra (SFTs), which are a type of vacancy cluster defect, are often formed. Therefore, SFTs were expected to form in the CoCrFeMnNi equiatomic high-entropy alloy (HEA). However, no SFT was observed in the CoCrFeMnNi HEA with high-speed plastic deformation even after annealing at 873 K. To elucidate this mechanism, the binding energy of vacancy clusters in the CoCrFeMnNi HEA was calculated based on first principles. The binding energy of the di-vacancy cluster was positive (average of 0.25 eV), while that of the tri-vacancy cluster was negative (average of − 0.44 eV), suggesting that the possibility of formation of a tri-vacancy cluster was low. The inability to form a cluster containing three vacancies is attributed to the excellent irradiation resistance of the CoCrFeMnNi HEA. However, if an extra vacancy is added to a tri-vacancy cluster (with negative binding energy), the binding energy of the subsequent tetra-vacancy cluster may become positive. This suggests that it is possible to form vacancy clusters in the CoCrFeMnNi HEA when high-energy ion or neutron irradiation causes cascade damage.

## Introduction

High-entropy alloys (HEAs) are composed of five or more elements with an equiatomic composition ratio, and the contribution of their configurational entropy to the Gibbs free energy is large. Therefore, a single-phase solid solution with a simple crystal structure, such as face-centered cubic (FCC), body-centered cubic (BCC), or hexagonal close-packed (HCP) structure, can be easily formed without the formation of intermetallic compounds^1^. The FCC single-phase solid solution CoCrFeMnNi equiatomic HEA was reported as the first example of an HEA^1^. This HEA has been widely studied, because it is stable alloy with excellent mechanical properties^2–8^.

**Figure 1:**
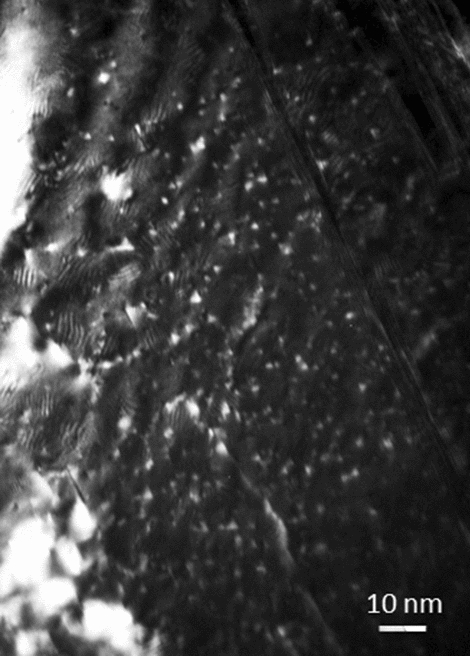
Stacking fault tetrahedra formed in Cu. The sample was deformed at room temperature with a strain rate of 10^6^/s. Dark-field weak-beam TEM observation along [110] direction.

It has been reported that the CoCrFeMnNi HEA has excellent irradiation resistance^9–12^. Although the reason for its good irradiation resistance is unclear, it is considered that the migration barrier of point defects induced by irradiation increases owing to the atomic-level stresses and local lattice distortions in the CoCrFeMnNi HEA. Investigation of the dependence of the irradiation dose and irradiation temperature on the microstructural evolution in the CoCrFeMnNi HEA is very important to determine the reasons for its excellent irradiation resistance. In addition to irradiation with high-energy particles, plastic deformation also produces point defects. Kiritani et al. reported that stacking fault tetrahedra (SFTs) with high density, a type of vacancy cluster defect, were formed during the plastic deformation of FCC metal (Au, Cu, and Ni) thin films at a strain rate of 10^5^/s–10^6^/s^13^. Among the point defects produced in the thin-film sample, the interstitials escape from the sample surface owing to their low migration energy. Therefore, high-density vacancies remain and form vacancy clusters. Because the stacking fault energies (SFEs) of FCC metals such as Au, Cu, and Ni (50, 55, and 250 mJ/m^2^, respectively) are low^14^, the SFT is formed by collapse of the vacancy cluster. Figure 1 shows an example of microstructures of a Cu thin film deformed at an estimated strain rate of 10^6^/s observed by dark-field weak-beam image of transmission electron microscopy (TEM) along the [011] direction with an operating diffraction of g = 200. The SFTs with an average size of 1 nm and density of approximately 2 × 10^24^/m^3^ were observed, resulting in a vacancy concentration of 2 × 10^–4^. Zaddach et al. reported that the SFE of CoCrFeMnNi HEA measured using X-ray diffraction and calculated based on first principles was approximately 25 mJ/m^2^^15^, which was lower than that of Ni. If the vacancies are clustered in the CoCrFeMnNi HEA, SFTs can be easily formed and observable using TEM.

In this study, well-annealed thin ribbon-shaped samples of the CoCrFeMnNi HEA were deformed at high speed, and its microstructures were observed using TEM. In addition, the microstructural evolution during annealing was investigated. The experimental results were discussed based on first-principles calculations.

## Results

**Figure 2:**
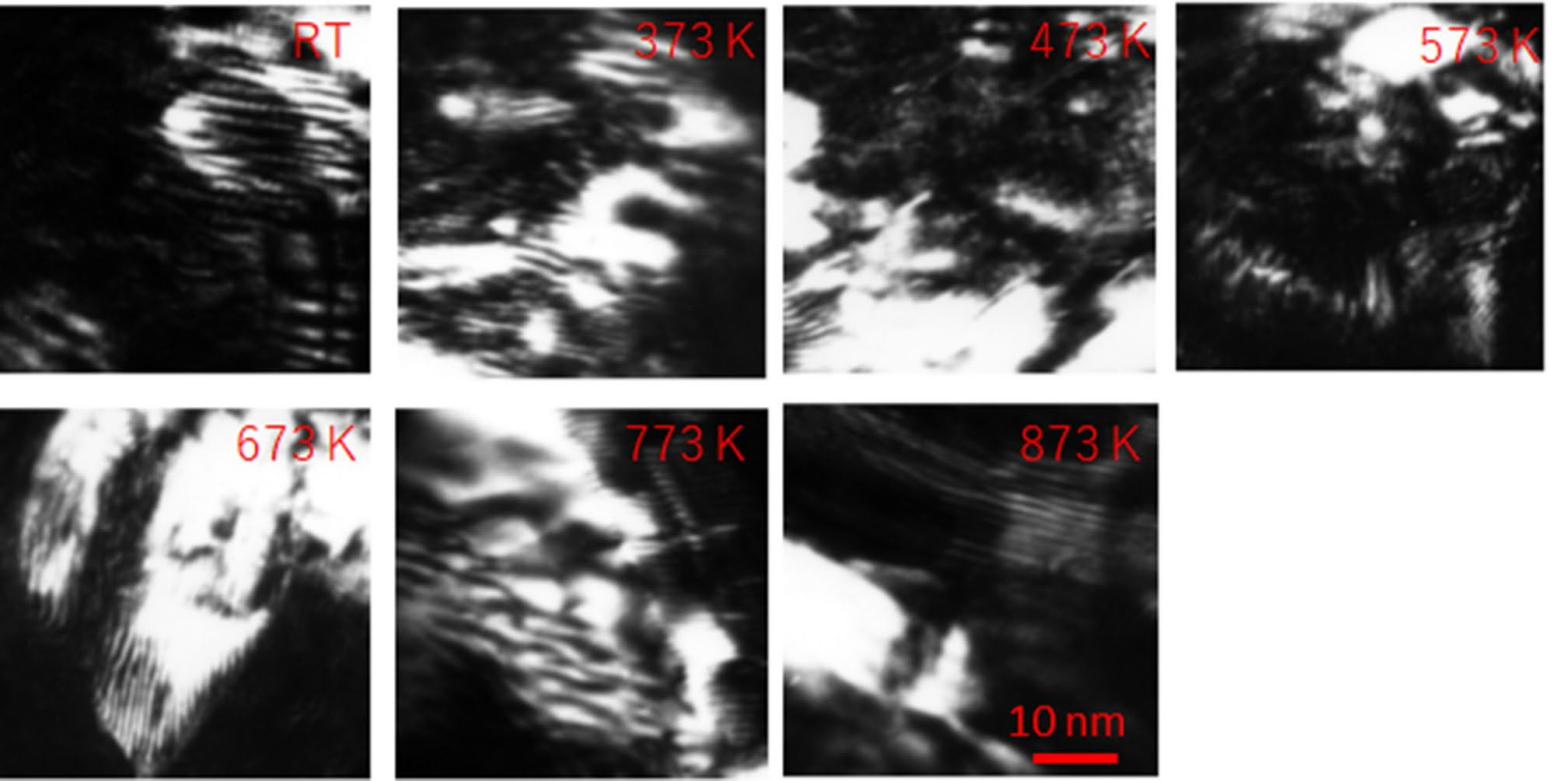
Microstructures in deformed CoCrFeMnNi. The sample was deformed at room temperature at a strain rate of 10^6^/s. Dark-field weak-beam TEM images of microstructures along [110] direction before and after annealing at various temperatures (room temperature to 873 K with increments of 100 K).

Figure 2 shows the dark-field weak-beam TEM images of the microstructures before (the upper-left corner figure) and after annealing the deformed CoCrFeMnNi HEA samples. After deformation, dislocations and twins were observed in the thicker central part of the sample. However, as shown in Fig. 1, the migration energy of the single vacancies in Cu was low (0.7 eV^16^) and they could move sufficiently at room temperature to form clusters, no SFTs (appearing as triangles) were observed on the thinner edges of the samples. Kiritani et al. proposed a model for vacancy formation during the high-speed heavy plastic deformation of metals. It was suggested that the vacancies were generated by the parallel shifting of a huge number of small areas of slip. In these small areas, the adjacent planes of atoms began to shift parallel to each other, and consequently, vacancies were formed^17^. It is thought that this model is applicable to the CoCrFeMnNi HEA. Using molecular dynamics simulation^18^, Do et al. reported that the migration energy of a single vacancy in the CoCrFeMnNi HEA was 0.79 ± 0.12 eV, although there were no reliable experimental data to support this calculation. Based on this result, if the jump frequency of a vacancy is assumed to be 10^13^/s, the vacancy diffusion in 10 min is over 200 nm at 473 K, which is much longer than the thinner edges of the samples of tens of manometers in size. The surface of the annealed sample was slightly oxidized by heating; consequently, the dark-field weak-beam images were not clear. However, no SFTs were observed even after annealing to 873 K. These experimental results showed that the formation of vacancy clusters was not possible in the CoCrFeMnNi HEA.

## Discussion

**Figure 3:**
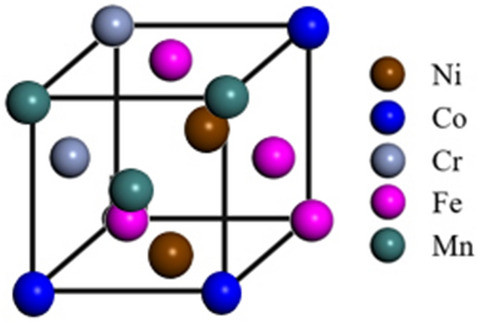
Atomic site of a perfect crystal with a uniform distribution of elements in the unit cell.

To understand the experimental results of the high-speed deformation, a simulation of the vacancy cluster formation in the CoCrFeMnNi HEA was performed using the first-principles energy calculations. Figure 3 schematically shows the site of a perfect crystal with a uniform distribution of elements in the supercell containing 180 atoms. Here, to create the (n + 1) vacancy cluster, only the 1 nn positions with respect to the n cluster were considered while removing an atom from the cell.

**Figure 4:**
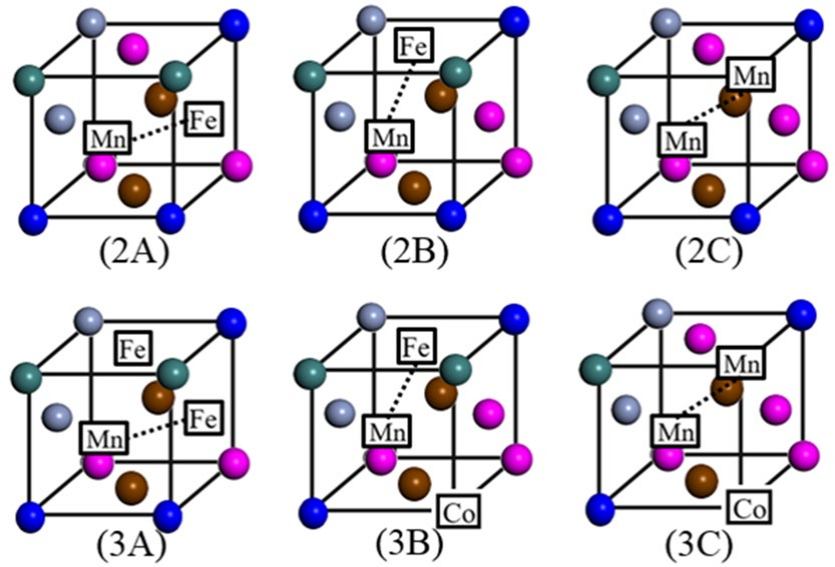
Configurations of the investigated di-vacancy (2A–2C) and tri-vacancy (3A–3C) clusters. The two elements enclosed within squares and connected by dotted lines in the figures (3A**–**3C) each indicate the configuration of the di-vacancy cluster formed first, and the other element (also enclosed within a square) represents the third vacancy of the tri-vacancy cluster.

As shown in Fig. 4, a single vacancy is generated by removing a Mn atom in the perfect crystal; subsequently, the atoms in the 1nn positions are selected to form di-vacancy (2A–2C) and tri-vacancy clusters (3A–3C), as shown in Fig. 3. The lower part of Fig. 4 shows three types of typical tri-vacancy cluster configuration^19^. The two elements enclosed within squares and connected by dotted lines in the figures (3A–3C) each indicate the configuration of the di-vacancy cluster formed first, and the other element (also enclosed within a square) represents the third vacancy of the tri-vacancy cluster. All three configurations were investigated for both clusters, as in the case of Cu^20^. Table 1 lists the binding energies of the vacancy clusters (N = 2, 3) in the CoCrFeMnNi HEA calculated using the above model. For both kinds of vacancy clusters, the binding energy depended on the arrangement of the vacancies. The binding energies of the di-vacancy cluster ranged between 0.08 and 0.39 eV, while those of the tri-vacancy cluster ranged between − 0.48 and − 0.35 eV. The positive binding energy indicated the tendency of the vacancy point defects to form di-vacancy clusters, which is a common phenomenon in traditional alloys. However, in case of the tri-vacancy clusters, the highly negative binding energies indicated that these clusters were unstable.

**Table 1:** Binding energies of di-vacancy and tri-vacancy clusters with different configurations.

	Binding energy (eV)	Configuration
(2A)	0.08	Mn + Fe
(2B)	0.39	Mn + Fe
(2C)	0.28	Mn + Mn
(3A)	− 0.35	Mn + Fe + Fe
(3B)	− 0.48	Mn + Fe + Co
(3C)	− 0.36	Mn + Mn + Co

**Figure 5:**
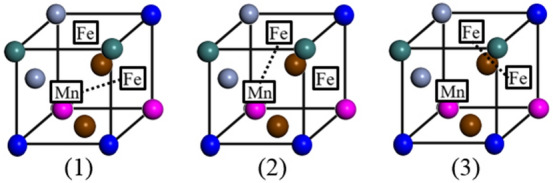
Schematic diagram of the 3A tri-vacancy cluster with different atomic vacancy formation sequences. The two elements enclosed within squares and connected by dotted lines in the figures each indicate the configuration of the di-vacancy cluster formed first, and the other element (also enclosed within a square) represents the third vacancy of the tri-vacancy cluster.

In our previous study on vacancy point defect formation energies^21^, the binding energy was quite different owing to different elemental environments. In this study, we investigated whether changing the vacancy formation sequence affected the atomic interaction. The binding energy of the 3A tri-vacancy cluster was more stable than those of the other two, because the vacancies were on the {111} planes of the FCC structure^22^. As shown in Fig. 5 and Table 2, the binding energies of the 3A tri-vacancy cluster with various atomic vacancy formation sequences were investigated; although there were some fluctuations in the absolute value, it was still negative.

**Table 2:** Binding energies of the 3A tri-vacancy cluster with different atomic vacancy formation sequences.

	Binding energy (eV)	Configuration
(1)	− 0.35	Mn + Fe + Fe
(2)	− 0.66	Mn + Fe + Fe
(3)	− 0.14	Fe + Fe + Mn

**Figure 6:**
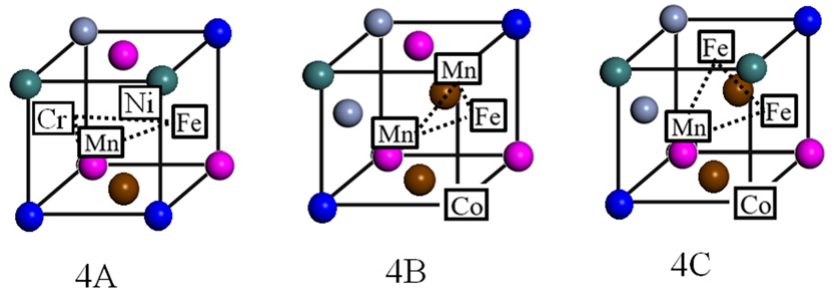
Schematic diagram of tetra-vacancy cluster composed of a single vacancy and a tri-vacancy with different atomic vacancy formation sequences. The three elements enclosed within squares and connected by dotted lines in the figures each indicate the configuration of the tri-vacancy cluster formed first, and the other element (also enclosed within a square) represents the fourth vacancy of the tetra-vacancy cluster.

**Figure 7:**
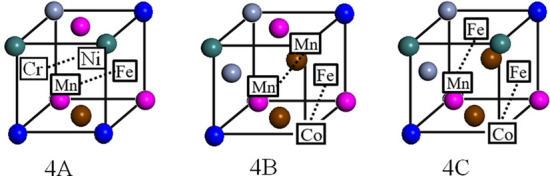
Schematic diagram of tetra-vacancy composed of two di-vacancies with different atomic vacancy formation sequences. The elements enclosed within squares and connected by dotted lines represent the configuration of the di-vacancy cluster formed first.

Generally, if the formation of tri-vacancy clusters is not possible, the formation of vacancy clusters containing more than three vacancies should also be impossible. However, in cascade damage, such as that caused by high-energy ion or neutron irradiation, vacancy clusters containing more than three vacancies can be formed. Furthermore, the formation of tetra-vacancy clusters is possible by the following combinations: a tri-vacancy cluster and a mobile single vacancy, and two mobile di-vacancies. Certainly, the probability of the latter is low. Therefore, it is important to investigate the binding energy of tetra-vacancy clusters. Figure 6 shows the configurations of the tetra-vacancy cluster in the CoCrFeMnNi HEA that appear to be stable^19^. The three elements enclosed within squares and connected by dotted lines in Fig. 6 each indicate the configuration of the tri-vacancy cluster formed first, and the other element (also enclosed within a square) represents the fourth vacancy of the tetra-vacancy cluster. The calculated binding energies are listed in Table 3. All the calculation results were positive. In addition, the configurations of the tetra-vacancy cluster (Fig. 7) were similar to those formed by two di-vacancies (Fig. 6). In Fig. 7, the elements connected by the dotted lines represent the configuration of the di-vacancies. Table 4 summarizes the binding energies, there are tetra-vacancy clusters with a positive binding energy. These results suggest that there are stable tetra-vacancy clusters in the CoCrFeMnNi HEA. In future, it is necessary to analyze in detail why the tetra-vacancy cluster is stable while the tri-vacancy cluster is unstable. At present, it is considered that the atomic size of different elements is different in the HEAs. For example, in the CoCrFeMnNi HEA, Co and Ni are undersized elements, while Cr, Fe, and Mn are oversized elements. The lattice sites in the HEAs are occupied randomly by the atoms of different elements, and this may introduce the large disturbance in the lattice structure. The instability of the tri-vacancy clusters is attributed to this lattice distortion. On the other hand, the tetra-vacancy clusters are stable due to the relaxation of lattice distortion.

**Table 3:** Binding energies of the tetra-vacancy cluster composed of a single vacancy and a tri-vacancy with different atomic vacancy formation sequences.

Tri-vacancy + single vacancy	Binding energy (eV)	Configuration
4 (A)	2.90	Fe + Ni + Cr + Mn
4 (B)	2.17	Fe + Co + Mn + Mn
4 (C)	2.38	Fe + Fe + Co + Mn

**Table 4:** Binding energies of the tetra-vacancy composed of two di-vacancies with different atomic vacancy formation sequences.

Two di-vacancy	Binding energy (eV)	Configuration
4 (A)	1.05	Fe + Ni + Cr + Mn
4 (B)	− 0.33	Fe + Co + Mn + Mn
4 (C)	0.73	Fe + Fe + Co + Mn

To provide a comparison with conventional metals, the binding energies of Vn (n = 2, 3, 4) in Ni, Cu with the same FCC structure were calculated. The average binding energy of Vn (n = 2, 3, 4) in the Ni, Cu, and CoCrFeMnNi HEA are listed in Table 5. In both Ni and Cu, the binding energies were positive, and increased with increasing size of the vacancy clusters. These calculation results were consistent with the findings of the growth of vacancy clusters in metals and alloys. However, it is impossible for the CoCrFeMnNi HEA to have tri-vacancy clusters. Previous studies^10,23^ have shown that HEAs can significantly improve irradiation resistance. The swelling of Ni can be effectively reduced by modifying the alloy composition to Ni-based HEAs^10^. The mean free path of electrons, phonons, and magnons can be reduced by the multi-chemical species in Ni-based HEAs, leading to energy dissipation and, consequently, affecting the defect evolution at early stages. Recently, using molecular dynamics simulations, Lin et al. investigated the generation and evolution of irradiation-induced defects in the CoCrFeNi HEA but not in the CoCrFeMnNi HEA. They concluded that the delayed damage accumulations in the CoCrFeNi HEA were attributed to the high defect recombination caused by the following: the enhanced thermal spike and low thermal conductivity for heat dissipation, and the substantially low binding energies of interstitial loops in the CoCrFeNi HEA^24^. However, the irradiation-tolerance mechanisms of Ni-based HEAs have remained unexplored and unclear owing to the multi-atomic interactions. In this study, the absence of a tri-vacancy cluster formation in the CoCrFeMnNi HEA can certainly suppress defect accumulation, which is different from that observed in traditional alloys. Limiting the formation of vacancy clusters in the CoCrFeMnNi HEA promoted the recombination of interstitials and vacancies formed by irradiation, and reduced the number of residual defect clusters, leading to the irradiation resistance.

**Table 5:** Average binding energies (eV) of vacancy clusters Vn (n = 2, 3, 4) in Ni, Cu, and CoCrNiFeMn HEA.

	Ni	Cu	CoCrNiFeMn
Di-vacancy	0.054	0.04	0.25
Tri-vacancy	0.164	0.06	− 0.38
Tetra-vacancy	0.18	0.11	1.48

Interstitials and vacancies are generated one by one when the sample undergoes tensile testing. Because there is no cascade damage, such as that caused by ion or neutron irradiation, it is difficult to form vacancy clusters containing four or more vacancies. Therefore, no vacancy clusters are observed in the CoCrFeMnNi HEA deformed at a high speed, as shown in Fig. 2. However, Jin et al. reported that voids were observed in the ion-irradiated CoCrFeMnNi HEA^10^. This contradictory to the present calculation results; however, in the case of ion irradiation, cascade damage directly forms the vacancy clusters containing four or more vacancies, and these clusters absorb vacancies to form voids. The simulations of cascade damage in the CoCrFeNi HEA have been carried out by Lin et al.^24^. They illustrated that the vacancy clusters containing more than three vacancies were formed by the cascades with primary knock-on (PKA) energies ranging from 10 to 50 keV. These results were in agreement with the result of this study, in which the tetra-vacancy cluster was found to be stable. Although the formation of vacancy clusters by the cascade depended on the PKA energy, the vacancy clusters containing more than three vacancies were about 10% of the surviving single vacancies formed by the cascade. The same calculations are required for the CoCrFeMnNi HEA. The cascade damages the materials to not only directly produce the large interstitial and vacancy clusters but also creates more surviving mobile vacancies in the material, because tiny interstitial clusters with high mobility disappear at the sinks, such as grain boundaries and precipitate surfaces. Excessive vacancies promote void swelling of the material and degrade the material^25^. Under cascade damage conditions, it was not possible to prevent the further growth of vacancy clusters containing more than three vacancies, according to the simulation results of the present study. However, the formation of vacancy clusters containing more than three vacancies from the state of excess vacancy, which were not directly formed by the cascades, was suppressed. Therefore, the irradiation resistance improves in the CoCrFeMnNi HEA even under cascade damage conditions such as ion and neutron irradiations.

In summary, the formation of vacancy clusters with respect to irradiation resistance was investigated in the CoCrFeMnNi HEA by experiments and theoretical calculations based on the first principles. After the high-speed deformation of the thin ribbon-shaped HEA sample, SFTs were not observed, unlike that in Cu with the same low SFE. SFTs did not appear even after annealing at 873 K. These results suggest that it is difficult to form vacancy clusters in the CoCrFeMnNi HEA. The simulation results showed the average binding energy of tri-vacancy was negative (− 0.44 eV), suggesting that they were unstable; however, there were tetra-vacancy clusters with positive binding energies. The inability to form tri-vacancy clusters is the main reason for the good irradiation resistance of the CoCrFeMnNi HEA.

## Methods

### Alloy fabrication, high-speed deformation experiment and microstructure observation

The CoCrFeMnNi equiatomic HEA was prepared in a vacuum induction furnace using high-purity (> 99.9%) elements such as Co, Cr, Fe, Mn, and Ni. A rectangular specimen (2 mm × 10 mm × 0.2 mm) was cut out of the homogenized ingot (in a vacuum at 1473 K for 10 h) and rolled to approximately 50 µm. The rolled sample was annealed in a vacuum of 10^–5^ Pa at 1273 K for 1 h. Electropolishing was performed using a HClO_4_ (25%) and CH_3_COOH (75%) solution to remove the oxide film formed on the sample surface by annealing. The electropolishing voltage was 15 V. The ends of this ribbon-shaped sample were held with pliers, and then torn off swiftly. The length of the thinned region was approximately 1 µm, and the deformation speed (corresponding to cross-head speed of the tensile testing) was 1 m/s. Therefore, the local strain rate was approximately 10^6^/s. The deformed sample was cut to a length of 2 mm and placed in a Cu mesh for TEM observation. Isochronal annealing experiments promoting the cluster of vacancies in this sample were carried out for 10 min in the temperature range between room temperature and 873 K, with increments of 100 K. The microstructures were observed by dark-field weak-beam image of TEM along the [011] direction of the FCC crystal with an operating diffraction of g = 200.

### Calculation of vacancy cluster binding energy

The density functional theory (DFT) was used to establish all models of this study. The calculations were performed using the Vienna ab-initio simulation package (VASP)^26,27^. The generalized gradient approximations (GGAs) parameterized by Perdew, Burke, and Emzerhof (PBE) were adopted to describe the exchange–correlation function^28^. The Brillouin zones were sampled using 3 × 3 × 2 K-points. The model structures were fully optimized using thresholds of 10^–4^ eV and 0.02 eV/A for the total energy and force, respectively. The electron wavefunctions for the alloy were expanded in the plane wave basis up to 400 eV. The preceding parameters were carefully selected through pre-calculation to obtain accurate results with an optimal set of computational values. The calculations were performed at a constant volume, and the atomic positions were relaxed using the conjugate gradient algorithm.

Point vacancy formation energies and chemical potentials of the various elements in the CoCrFeMnNi HEA were calculated in our previous study^21^. The formation energy of a cluster containing n vacancies, E^f^_N_, is expressed by the following equation:

1 $$ {\text{E}}^{{\text{f}}}_{{\text{N}}} = {\text{ E}}_{{{\text{defect}}}} {-} \, ({\text{E}}_{{{\text{perfect}}}} - {{\upmu}}_{{\text{p}}} ) , $$

where E_defect_ and E_perfect_ are the energies with and without vacancies, respectively. *µ* is the chemical potential of the atom, and *p* is the type of atom (Co, Cr, Fe, Mn, Fe).

The binding energy E(b) of a cluster with vacancy number n (n = 2, 3, 4) is expressed by the following equation:

2 $$ {\text{E}}\left( {\text{b}} \right) \, = {\text{ E}}^{{\text{f}}} \left( {\text{a}} \right) \, + \, \left( {{\text{E}}^{{\text{f}}} \left( {\text{b}} \right) \, {-}{\text{ E}}^{{\text{f}}} \left( {{\text{ab}}} \right)} \right), $$

where, E^f^(a), E^f^(b), and Ef(ab) are the formation energies of a single vacancy, the (n − 1) vacancy, and the n vacancy clusters, respectively. A positive binding energy indicates attraction between the defects. To create the (n + 1) vacancy cluster, only the first nearest- neighbor (1nn) positions with respect to the n cluster were considered while removing an atom from the cell.

The CoCrFeMnNi HEA is a 3 × 3 × 5 supercell with a total equivalent atomic number of 180. The structural model and optimization of atomic arrangements were the same as those in our previous study^21^, where the supercell structure was constructed using a similar atomic environment (SAE) method and an efficient Widom-type approach^29–31^. Figure 3 schematically shows the site of a perfect crystal with a uniform distribution of elements in the supercell containing 180 atoms. Here, to create the (n + 1) vacancy cluster, only the 1 nn positions with respect to the n cluster were considered while removing an atom from the cell.

## References

[1] Yeh JW, Chen SK, Gan JY, Lin SJ, Chin TS, Shun TT, Tsau CH, Chang SY (2004). Formation of simple crystal structures in CuCoNiCrAlFeTiV alloys with multiprincipal metallic elements. Metall. Mater. Trans. A.

[2] Yeh JW, Chen SK, Lin SJ, Gan JY, Chin TS, Shun TT, Tsau CH, Chang SY (2004). Nanostructured high-entropy alloys with multiple principal elements: Novel alloy design concepts and outcomes. Adv. Eng. Mater..

[3] Miracle DB, Miller JD, Senkov ON, Woodward C, Uchic MD, Tiley J (2014). Exploration and development of high entropy alloys for structural applications. Entropy.

[4] Gludovatz B, George EP, Ritchie RO (2015). Processing, microstructure and mechanical properties of the CrMnFeCoNi high-entropy alloy. JOM..

[5] Tsai MH, Yeh JW (2014). High-entropy alloys: a critical review. Mater. Res. Lett..

[6] Cantor B, Chang ITH, Knight P, Vincent AJB (2004). Microstructural development in equiatomic multicomponent alloys. Mater. Sci. Eng. A.

[7] Gali A, George EP (2013). Tensile properties oh high- and medium-entropy alloys. Intermetallics.

[8] Pickering EJ, Jones NG (2016). High-entropy alloys: A critical assessment of their founding principles and future prospects. Int. Mater. Rev..

[9] Li YE, Li R, Peng Q (2020). Enhanced surface bombardment resistance of the CoNiCrFeMn high entropy alloy under extreme irradiation flux. Nanotechnology.

[10] Jin K, Lu C, Wang LM, Qu J, Weber WJ, Zhang Y, Bei H (2016). Effects of compositional complexity on the ion-irradiation induced swelling and hardening in Ni-containing equiatomic alloys. Scr. Mater..

[11] Yang LX, Ge HL, Zhang J, Xiong T, Jin QQ, Zhou YT, Shao XH, Zhang B, Zhu ZW, Zheng SJ, Ma XL (2019). High He-ion irradiation resistance of CrMnFeCoNi high-entropy alloy revealed by comparison study with Ni and 303SS. J. Mater. Sci. Technol..

[12] Ren XL, Zhong ZH, Zhu T, Yao BD, Wang YX, Cao XZ, Jinno S, Xu Q (2020). Effect of irradiation on randomness of element distribution in CoCrFeMnNi equiatomic high-entropy alloy. Intermetallics.

[13] Kiritani M, Yasunaga K, Matsukawa Y, Komatsu M (2002). Plastic deformation of metal thin films without involving dislocations and anomalous production of point defects. Rad. Eff. Def. Sol..

[14] Physical metallurgy, Fourth, revised and enhanced edition, (ed. Cahn, W. & Haasen, P.) 191.

[15] Zaddach AJ, Niu C, Koch CC, Irving DL (2013). Mechanical properties and stacking fault energies of NiFeCrCoMn high-entropy alloy. JOM.

[16] Balluffi RW (1978). Vacancy defect mobilities and binding energies obtained from annealing studies. J. Nucl. Mater..

[17] Kiritani M, Satoh Y, Kizuka Y, Arakawa K, Ogasawara Y, Arai S, Shimomura Y (1999). Anomalous production of vacancy clusters and the possibility of plastic deformation of crystalline metals without dislocations. Philos. Mag. Lett..

[18] Do HS, Lee BJ (2018). Origin of radiation resistance in multi-principal element alloys. Sci. Rep..

[19] Sabochick MJ, Yip S (1988). Migration energy calculations for small vacancy clusters in copper. J. Phys. F: Met. Phys..

[20] Song H, Tian F, Qing-Miao Hu, Vitos L, Wang Y, Shen J, Chen N (2017). Local lattice distortion in high-entropy alloys. Phys. Rev. Mater..

[21] Guan HQ, Huang SS, Ding JH, Tian FY, Xu Q, Zhao JJ (2020). Chemical environment and magnetic moment effects on point defect formations in CoCrNi-based concentrated solid-solution alloys. Acta Mater..

[22] Was, G.S. *Fundamentals of Radiation Materials Science*. (SpringerNature, New York).

[23] Lu C, Jin K, Béland LK, Zhang F, Yang T, Liang Q, Zhang Y, Bei H, Christen HM, Stoller RE (2016). Direct observation of defect range and evolution in ion-irradiated single crystalline Ni and Ni binary alloys. Sci. Rep..

[24] Lin Y, Yang T, Lang L, Shan C, Deng H, Wangyu Hu, Gao F (2020). Enhanced radiation tolerance of the Ni-Co-Cr-Fe high-entropy alloy as revealed from primary damage. Acta Mater..

[25] Singh BN, Zinkle SJ (1993). Defect accumulation in pure fcc metals in the transient regime: a review. J. Nucl. Mater..

[26] Kresse G (1995). Ab initio molecular dynamics for liquid metals. J. Non-Cryst. Solids.

[27] Kresse G, Hafner J (1994). Ab initio molecular-dynamics simulation of the liquid-metal-amorphous-semiconductor transition in germanium. Phys. Rev. B.

[28] Kresse G, Furthmuller J (1996). Efficiency of ab-initio total energy calculations for metals and semiconductors using a plane-wave basis set. Comput. Mater. Sci..

[29] Cantor B, Chang ITH, Knight P, Vincent AJB (2004). Microstructural development in equiatomic multicomponent alloys. Mater. Sci. Eng..

[30] Lucas MS, Mauger L, Munoz JA, Xiao YM, Sheets AO, Semiatin SL, Horwath J, Turgut Z (2011). Magnetic and vibrational properties of high-entropy alloys. J. Appl. Phys..

[31] Tian, F., Lin, D., Cao, X., Song, H., Zhao, Y., Song, H. A structural modeling approach to the solid-solution materials, *arXiv preprint* **1810**, 06144 (2018).

